# CT texture analysis as a prognostic marker in metastatic colorectal cancer patients treated with bevacizumab

**DOI:** 10.1186/1470-7330-15-S1-S12

**Published:** 2015-10-02

**Authors:** Shih-Hsin Chen, Julien Edeline, Kai-Keen Shiu, Sarah Benafif, Sofia Wong, Ashley Groves, John Bridgewater, Balaji Ganeshan

**Affiliations:** 1 University College London, Gower Street, London, WC1E 6BT, UK

## Aim

Following anti-angiogenic treatment, response might be represented by changes in tumour heterogeneity and may not be reflected on traditional size-based criteria. CT texture analysis (CTTA) is one emerging tool to quantify tumour heterogeneity, and has been shown to be prognostic in different tumour applications. We aimed to assess the association of CTTA with overall survival (OS) in a series of metastatic colorectal cancer (mCRC) patients treated with bevacizumab.

## Methods

We retrospectively gathered clinical and imaging data from mCRC patients treated with bevacizumab plus chemotherapy from 3 centres in the UK. CTTA comprised the image filtration-histogram technique using commercially available TexRAD research software platform (TexRAD ltd http://www.texrad.com, part of Feedback Plc).

## Results

101 patients were identified, of which 67 patients had both pre-treatment and first post-treatment CT scans available for image analysis and response evaluation. 38 patients were treated in first line, 29 in latter lines. Median OS was 12.4 months. Several texture parameters were significantly associated with OS at pre- and post-treatment scans, including mean (p=0.001), mean of positive pixels (p=0.002), entropy (p=0.001) and kurtosis (p=0.004). Furthermore change in entropy between post- and pre-treatment scans was significantly associated with OS (p=0.009) with an increase in post-treatment entropy (>0.1) associated with worse outcome. Particularly post-treatment entropy > 4.51 was the best univariate marker of survival (HR: 3.0, 95% CI = 1.5-5.9, p=0.001).

## Conclusion

This retrospective study highlighted the potential of CTTA to be a prognostic marker in mCRC patients treated with bevacizumab and chemotherapy.

